# The effect of trait mindfulness on rumination in college students: the mediating role of physical exercise behavior and optimistic intelligence quotient

**DOI:** 10.3389/fspor.2026.1645772

**Published:** 2026-03-13

**Authors:** Yong Jiang, Beier Zhang, Hongbo Zhao, Lei Shi

**Affiliations:** 1 School of Physical Education, Liaoning Normal University, Dalian, China; 2 School of Physical Education, Shenyang Medical College, Shenyang, China

**Keywords:** exercise behaviour, optimistic intelligence quotient, rumination, sport psychology, trait mindfulness

## Abstract

**Objective:**

To explore the mechanism of trait mindfulness's influence on college students' rumination and to analyze the chain mediating role of exercise behavior and optimistic intelligence quotient.

**Methods:**

A questionnaire survey was conducted on 1983 Chinese university students (mean age of subjects 19.10 ± 1.07 years) using the Trait mindfulness Scale, the Rumination Scale, the Physical Exercise Rating Scale, and the Optimization Scale. Among them, 996 were male and 987 were female university students.

**Results:**

The direct effect value of trait mindfulness on college students' rumination was −0.013, the effect values of exercise behavior and music quotient in the relationship between trait mindfulness and college students' rumination were −0.005 and −0.003, respectively, and the chain mediation effect of exercise behavior and music quotient was significant with an effect value of −0.093.

**Conclusions:**

(1) trait mindfulness significantly and positively predicts exercise behavior and music quotient, and significantly and negatively predicts rumination among college students; (2) trait mindfulness has significant direct and indirect effects on rumination among college students. trait mindfulness can significantly predict rumination through the independent mediating effect of exercise behavior and music quotient, and can also significantly predict rumination through the chain mediating effect of exercise behavior and music quotient.

## Introduction

As the driving force behind future socioeconomic development, college students face significant challenges affecting their mental health. This has, to some extent, led to a gradual deterioration in their mental state, manifesting as rumination (1). Rumination refers to the repetitive, passive preoccupation with negative events, one's own negative emotional state, and the potential causes and consequences of such events following a negative experience (2). This cognitive pattern has garnered extensive attention in psychology, neuroscience, and brain science, as it not only correlates with various mental health issues but also profoundly impacts individuals' emotional regulation, cognitive functions, and overall health. Among the factors influencing rumination in college students, trait mindfulness stands as a crucial predictor of mental health. It can be cultivated through mindfulness meditation practice, which directs attention toward the present moment rather than dwelling on past “memories” (3). In recent years, trait mindfulness has garnered widespread recognition across disciplines such as sociology and sports psychology for its positive role in self-emotional regulation and mental health maintenance (4). Research indicates that trait mindfulness can reduce rumination by directing attention to breathing and the present moment (5). Individuals who engage in eight weeks of mindfulness practice exhibit significantly reduced activity in the default mode network (DMN), which is associated with “mind wandering”. Overactivity in the DMN can trigger unconscious distractions, rumination, and emotional exhaustion. Mindfulness practice, by focusing on present-moment experiences like breathing and bodily sensations, gently guides wandering thoughts back to the present. This gradually enhances the brain's attentional control, preventing automatic playback of chaotic thoughts and thereby reducing rumination. This study focuses on Chinese university students as subjects. Due to the high difficulty of contemporary university courses and intense academic demands, their rumination levels are significantly higher than those of other groups. Previous research has rarely delved into the rumination patterns of this specific professional cohort, and this study aims to fill this gap. Therefore, this study aims to explore the relationship and underlying mechanisms between trait mindfulness and rumination among college students. For the first time, it incorporates exercise behaviour and optimistic intelligence quotient (JOQ), providing theoretical foundations and practical guidance for reducing rumination among this demographic.

## Research hypothesis

### The relationship between trait mindfulness and rumination in college students

Trait mindfulness refers to an individual's habitual awareness of present thoughts and emotions, encompassing observing experiences, labeling perceptions verbally, maintaining present-moment awareness, refraining from judging one's thoughts and feelings, and avoiding overreaction (6). According to the trait mindfulness coping model, when individuals encounter stressful events, trait mindfulness enhances cognitive flexibility, enabling them to respond through positive reappraisal (7). Therefore, when confronting stressful events, individuals with high trait mindfulness allocate more cognitive resources to maintain present-moment awareness, alter existing cognitive patterns (8), and reappraise past experiences positively (9), thereby shifting rumination from negative to positive (10). Since the 1970s, numerous studies have confirmed the efficacy of mindfulness training in alleviating social anxiety and depression. Mindfulness practice effectively treats various psychological disorders, offering significant benefits for rumination, anxiety disorders, and depression. Trait mindfulness helps college students reduce rumination primarily by using mindful meditation to detach attention from repetitive thoughts and redirect it toward present-moment experiences. Research indicates a negative correlation between trait mindfulness and rumination (11), enhances individuals' executive control functions (12), and positively impacts mental health by reducing rumination, increasing psychological flexibility, and boosting overall well-being through heightened mindfulness. Attention Control Theory posits that trait mindfulness enhances an individual's ability to manage attentional resources, while deficits in attention control are a key trigger for rumination. College students are prone to experiencing attentional dispersion or becoming “trapped in negative thoughts” due to external distractions (such as social media notifications or multiple academic tasks) or internal negative thoughts—precisely the foundation for rumination to occur. Through training in “anchoring attention (e.g., focusing on breathing), noticing distractions, and refocusing attention”, trait mindfulness enhances individuals' attentional stability and shifting abilities. This enables them to proactively redirect attention from negative ruminative content to present tasks or neutral information, thereby reducing the duration and intensity of rumination. Given this, this study proposes Hypothesis H1: Trait mindfulness has a negative predictive effect on rumination in college students.

### Mediating role of exercise behavior

Exercise behaviour refers to physical activities voluntarily chosen by individuals based on their needs, employing various sports methods combined with natural forces and hygiene measures (13). As a positive personality trait, the two core elements of trait mindfulness—“present-moment awareness” and “non-judgmental attitude”—can to some extent motivate college students to persist in exercise, thereby fostering consistent physical activity (14). Research indicates a significant positive correlation between trait mindfulness and exercise levels. Individuals with high trait mindfulness are more likely to maintain regular physical activity, which helps reduce rumination (15). Specifically, it positively correlates with sustaining physical activity, achieving exercise goals, and satisfaction with physical activity (16). Higher mindfulness levels among college students imply greater likelihood of long-term exercise persistence (17). In recent years, numerous scholars have increasingly focused on the impact of physical exercise on rumination. Research reveals a close relationship between physical exercise and rumination, with exercise exerting a positive influence on rumination. Brand et al. suggest that physical exercise of varying intensities can alleviate rumination among college students, with moderate-intensity exercise proving most effective in reducing rumination (18). Given this, this study proposes Hypothesis H2: exercise behaviour mediates the relationship between trait mindfulness and rumination.

### The mediating role of optimistic intelligence quotient

Seligman's learned optimism theory posits that personality is divided into “optimistic explanatory styles” and “pessimistic explanatory styles”. Individuals with optimistic explanatory styles typically view failures and setbacks as temporary, caused by specific situational events and external factors, and confined to the immediate context. In contrast, individuals with a pessimistic explanatory style attribute failures and setbacks to long-term or permanent, universal, and personal internal causes, believing these will generalize to other matters. The concept of optimistic intelligence quotient (OIQ) extends and expands upon this explanatory style theory to some extent, emphasizing that individuals not only possess optimistic cognitive tendencies but also have the capacity to derive joy from both positive and negative events, thereby enhancing their levels of optimism (19). Research indicates that trait mindfulness significantly and positively predicts optimistic intelligence quotient (20), correlates positively with individual optimism (21), and facilitates the development of positive emotional regulation strategies, enabling individuals to remain more optimistic and confident when confronting negative events (22). Furthermore, optimistic intelligence quotient can reduce rumination when individuals face negative life events (23). Research indicates that optimistic intelligence quotient can tap into internal positive resources when confronting negative life events, actively coping with stress to adapt to the environment. Simultaneously, it enables individuals to effectively release negative emotions inwardly through reasonable means and outwardly seek help to build effective social support (24). Given this, this study proposes Hypothesis H3: Optimistic intelligence quotient mediates the relationship between trait mindfulness and rumination.

### Chain mediation effect of trait mindfulness on rumination

Physical exercise demonstrates positive effects across various domains, with positive emotions primarily derived from such activities. Exercise behaviour positively influences college students' levels of optimistic intelligence quotient (25), and related variables (frequency, duration, intensity) significantly predict their optimistic intelligence quotient (26). Research indicates that individuals with higher exercise behaviour exhibit greater optimistic intelligence quotient (27), and university students' optimistic intelligence quotient levels increase with longer exercise duration and higher frequency (28). Regular physical exercise effectively improves mood states, promotes the adoption of positive coping strategies, and mitigates stress-induced harm (29). Self-Determination Theory explains this relationship through the lens of motivation and psychological need fulfillment. Physical exercise satisfies three fundamental psychological needs: autonomy, competence, and relatedness. Fulfilling these needs enhances intrinsic motivation, making individuals more inclined to actively seek positive experiences and cope with setbacks. This tendency toward proactive mental regulation constitutes a core component of optimistic intelligence quotient. Thus, regular exercise provides an internal driving force for enhancing optimistic intelligence quotient by fulfilling psychological needs. After physical exercise, college students often more readily attain positive subjective experiences such as subjective well-being, euphoria from exercise, a sense of accomplishment, enjoyment of physical activity, positive interpersonal relationships, and social support. These positive subjective experiences are all manifestations of high optimistic intelligence quotient (30). Based on this, this study proposes Hypothesis H4: Exercise behaviour mediates the relationship between trait mindfulness and optimistic intelligence quotient in a chain-like manner.

**Figure 1:**
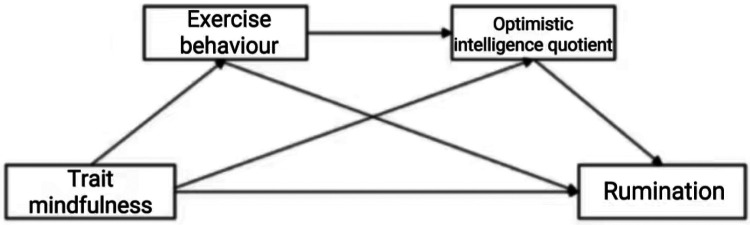
Hypothesized model of trait mindfulness affecting rumination in college students.

In summary, to examine the influence of trait mindfulness on rumination among college students, this study proposes to construct a chain mediation model (as shown in Figure 1) and will test the following aspects: (1) Trait mindfulness significantly and negatively predicts rumination in college students; (2) Exercise behaviour and optimistic intelligence quotient exert separate mediating effects between trait mindfulness and rumination in college students; (3) Exercise behaviour and optimistic intelligence quotient mediate the relationship between trait mindfulness and rumination in college students through a chained mediation effect.

## Research participants and methods

### Participants and sampling

Using convenience sampling, an online questionnaire survey was conducted from September 1 to September 20, 2024, among 2,024 college students from 15 universities in Liaoning and Henan provinces, China. The survey employed the Trait Mindfulness Scale, Rumination Scale, Exercise Behaviour Level Scale, and Optimistic Intelligence Quotient Scale. Liaoning and Henan were selected due to established collaborative relationships and their large, diverse student populations. During questionnaire screening, 20 responses with inconsistent answers to reverse-scored items, 11 responses completed in excessively short time frames, and 10 responses showing systematic errors were excluded. Ultimately, 1,983 valid questionnaires were recovered, yielding a response rate of 97.9%. Among the respondents, 996 were male (49.8%) and 987 were female (50.2%), with an average age of 19.10 ± 1.07 years. All 1,983 participants actively engaged in exercise behaviour and demonstrated understanding of the concepts of trait mindfulness, rumination, and optimistic intelligence quotient.

### Research tools

#### Trait mindfulness scale

The trait mindfulness scale (31), translated and revised by Chen Siyi et al., was adopted. This 15-item scale includes statements such as “When I am on my way to a destination, I do not pay attention to other things along the way”. Scoring uses a 6-point Likert scale ranging from “almost always” (1) to “almost never”(6). Higher scores indicate greater mindfulness levels and stronger present-moment attention and awareness. This scale was selected for its status as a classic tool for measuring trait mindfulness, effectively capturing stable individual tendencies rather than transient states. Its brevity also suits large-scale administration. The Cronbach's α coefficient for this scale is 0.895.

#### Rumination scale

The rumination scale translated and revised by Han Xiu et al. (32) was employed. This 22-item scale comprises three dimensions: symptom rumination, reflective rumination, and obsessive rumination (focused on repeated thinking about physical or psychological symptoms themselves, rational and purposeful deep reflection on problems or experiences, and recurrent intrusive negative thoughts or impulses). Items describe individual preoccupations with self, depressive symptoms, causes, and consequences. Scoring uses a 4-point Likert scale (1 = Never, 4 = Always), with higher total scores indicating greater rumination levels. This scale was selected for the study due to its multidimensional structure, which allows for precise differentiation of rumination components. This facilitates in-depth analysis of mindfulness's differential mechanisms across these components. The scale's Cronbach's α coefficient is 0.939.

#### Physical exercise level scale

The Physical Exercise Level Scale, revised by Liang Deqing et al. (33), assesses exercise volume by measuring three dimensions: exercise intensity, duration per session, and weekly frequency. It demonstrates high reliability and validity. The scale uses a 5-point Likert scale, with higher total scores indicating better exercise behaviour. Scoring is calculated as Exercise Volume = Intensity × (Time - 1) × Frequency, ranging from a minimum of 0 to a maximum of 100 points. This scale was selected for the study due to its clear operational definitions, objective scoring method, and ability to quantitatively grade exercise behaviour. This facilitates analysis of dose-response relationships with psychological variables and makes it highly suitable for assessing activity patterns among college students. The scale's Cronbach's α coefficient is 0.875.

#### Optimistic intelligence quotient scale

The Optimistic Intelligence Quotient Scale developed by Ren Jun (34) was adopted. This 18-item scale comprises four dimensions: Optimism Index, Appreciation Ability, Negative Event Coping Ability, and Joy Contagion Ability (measuring the stability of positive expectations toward future events, the psychological capacity to actively perceive, appreciate, and prolong positive experiences, cognitive and coping abilities when facing negative events, and the ability to transmit positive emotions to others through social interactions). The scale employs a 4-point Likert scoring system, ranging from “Not at all like me” (1 point) to “Very much like me” (4 points). Higher total scores indicate greater optimistic intelligence quotient. This localized scale was selected for the study to more accurately measure comprehensive joy capabilities rooted in Chinese cultural psychology. Its multidimensional structure comprehensively reflects positive psychological qualities, with an internal consistency reliability Cronbach's α coefficient of 0.872.

#### Data statistics and analysis

Data analysis was conducted using SPSS 27.0 with the Process plugin, including internal consistency testing, Pearson correlation coefficient analysis, regression analysis, and bias-corrected Bootstrap method testing for the trait mindfulness scale, rumination scale, exercise behaviour level scale, and optimistic intelligence quotient scale. The Bootstrap method employed a sample size of 5,000 with a 95% confidence interval. An effect was considered significant if the confidence interval excluded zero, and vice versa. Additionally, the G*Power software was used to calculate the required sample size for the questionnaire survey. With an effect size of 0.30, significance level of 0.05, and statistical power of 0.80, the calculated sample size was 82. The final sample size for this study was 1,983, significantly exceeding the 82 required by G*Power analysis. This decision was made to enhance statistical power and precision. A larger sample size allows estimates to more closely approximate the true population parameter values, thereby reducing sampling error.

## Research findings

### Common method bias test

To mitigate the impact of common method bias on research outcomes, necessary controls were implemented during data collection, such as requiring anonymous responses from participants and employing reverse scoring for certain items. The Harman single-factor test was applied to assess common method bias. Results indicated nine factors with eigenvalues exceeding 1. The first factor explained 27.1% of variance, significantly below the 40% critical threshold (36), confirming no common method bias in the data.

### Descriptive statistics and correlation analysis of variables

Independent samples *t*-tests were conducted to analyze gender differences in trait mindfulness, rumination, exercise behaviour, and optimistic intelligence quotient. Results revealed that male college students significantly outperformed female students in trait mindfulness, rumination, and exercise behaviour. Conversely, female students demonstrated significantly higher optimistic intelligence quotient than male students (see Table 1).

**Table 1:** Gender differences between different variables.

Variable	Genders	Number	M ± SD	*F*	*P*
Trait mindfulness	Male	996	3.83 ± 1.17	5.102	0.024*
	Female	987	3.71 ± 1.18		
Rumination	Male	996	1.41 ± 0.36	0.206	0.650
	Female	987	1.41 ± 0.42		
Exercise behaviour	Male	996	3.94 ± 0.78	0.881	0.348
	Female	987	3.91 ± 0.75		
Optimistic intelligence quotient	Male	996	3.24 ± 0.26	3.691	0.000**
	Female	987	3.50 ± 0.38		

*N* = 1,983.

* *p* < 0.05.

** *p* < 0.01.

*** *p* < 0.001.

A one-way ANOVA was conducted to compare differences in trait mindfulness, rumination, exercise behaviour, and optimistic intelligence quotient across different grade levels. The results revealed significant differences in trait mindfulness, rumination, exercise behaviour, and optimistic intelligence quotient among college students of various grades. Overall, upperclassmen exhibited higher trait mindfulness than underclassmen; freshmen exhibited the lowest rumination levels, while sophomores demonstrated the highest; freshmen showed the strongest exercise behaviours, whereas juniors displayed the weakest; sophomores had the weakest exercise behaviours, while juniors exhibited the strongest (see Table 2).

**Table 2:** Differences in grade levels between variables.

Variable	Grade	Number	M ± SD	*F*	*P*
Trait mindfulness	Freshman	499	3.99 ± 1.19	7.819	0.000**
	Sophomore	559	3.69 ± 1.14		
Rumination	Junior	496	3.68 ± 1.12	121.029	0.000**
	Senior	429	3.76 ± 1.24		
	Freshman	499	1.23 ± 0.32		
	Sophomore	559	1.61 ± 0.30		
Exercise behaviour	Junior	496	1.47 ± 0.42	3.904	0.000**
	Senior	429	1.29 ± 0.40		
	Freshman	499	4.01 ± 0.83		
	Sophomore	559	3.88 ± 0.72		
Optimistic intelligence quotient	Junior	496	3.86 ± 0.78	145.578	0.000**
	Senior	429	3.96 ± 0.71		
	Freshman	499	3.34 ± 0.28		
	Sophomore	559	3.17 ± 0.22		
	Junior	496	3.57 ± 0.25		
	Senior	429	3.42 ± 0.48		

*N* = 1,983.

* *p* < 0.05.

** *p* < 0.01.

*** *p* < 0.001.

Pearson correlation analysis was conducted on all variables to validate the potential relationships among trait mindfulness, rumination, exercise behaviour, and optimistic intelligence quotient. As shown in Table 3, significant negative correlations were found between trait mindfulness, exercise behaviour, and optimistic intelligence quotient (*p* < 0.01). This indicates that higher levels of trait mindfulness are associated with stronger exercise behaviour and optimistic intelligence quotient, while lower levels of rumination are observed, thereby supporting Hypothesis H1. The strong statistical significance of these correlations indicates suitability for subsequent mediation analysis and supports further hypothesis testing.

**Table 3:** Correlation analysis between variables.

Variable	M	SD	1	2	3	4
Trait mindfulness	3.774	1.176	1			
Rumination	1.410	0.392	−0.062**	1		
Exercise behaviour	3.928	0.766	0.185**	−0.090**	1	
Optimistic intelligence quotient	3.368	0.349	0.103**	−0.091**	0.048*	1

*N* = 1,983.

* *p* < 0.05.

** *p* < 0.01.

*** *p* < 0.001.

Due to significant correlations among all variables, multicollinearity may exist, potentially leading to unstable results. (Multicollinearity refers to the strong linear relationship between two or more independent variables in a multiple linear regression model, resulting in inaccurate parameter estimates and reduced explanatory power.) Therefore, this study conducted collinearity diagnostics and standardized (Z-scored) all predictor variables in each subsequent equation. Results indicate that all variance inflation factors (VIF) for predictor variables (ranging from 1.439 to 4.100) are below 5. Thus, the data exhibit no multicollinearity issues, meeting the conditions for further chained mediation effect testing.

Since trait mindfulness, rumination, exercise behaviour, and optimistic intelligence quotient all showed statistically significant correlations (*P* < 0.01), they met the criteria for mediation effect testing. Using trait mindfulness as the independent variable, rumination as the dependent variable, and introducing exercise behaviour and optimistic intelligence quotient as mediating variables, we examined whether trait mindfulness exerted a significant mediating effect and chain mediating effect on exercise behaviour. This study employed the PROCESS macro program for SPSS provided by Hayes (37), utilizing Model 6 (mediation model) with grade and gender as control variables to test the chain mediation model effects. Bootstrap samples were set to 5,000 with a default 95% confidence interval; results were considered significant when the confidence interval did not include zero.

Results indicate that before including mediators, trait mindfulness significantly predicted rumination (*β* = −0.185, *t* = −2.750, *p* < 0.001). After including the mediating variable, the predictive effect of trait mindfulness→rumination remained significant (*β* = −0.062, *t* = −1.693, *p* < 0.001). Specifically, the path coefficient (β) for trait mindfulness→exercise behaviour was 0.120, exercise behaviour→ rumination was 0.267, trait mindfulness→optimistic intelligence quotient was 0.031, optimistic intelligence quotient→rumination was 0.412, and exercise behaviour→ optimistic intelligence quotient was 0.015 (Table 4).

**Table 4:** Regression analysis of trait mindfulness, exercise behaviour, and optimistic intelligence quotient on rumination.

Outcome variable	Predictor variable	Goodness-of-fit indicator			Significance of coefficients	
		R	R^2^	F	*β*	*t*
Exercise behaviour	Grade	0.185	0.034	23.472***	−0.011	−0.351
	Genders				0.002	0.032
	Trait mindfulness				0.120	8.305***
Optimistic intelligence quotient	Grade	0.049	0.202	124.952***	−0.138	−10.857
	Genders				0.522	18.909
	Trait mindfulness				0.031	5.149***
	Exercise behaviour				0.015	1.618***
Rumination	Grade	0.228	0.052	21.751***	−0.030	−0.616
	Genders				0.163	−1.478
	Trait mindfulness				−0.105	−1.588***
	Exercise behaviour				0.267	7.883***
	Optimistic intelligence quotient				0.412	5.012***

*N* = 1,983.

* *p* < 0.05.

** *p* < 0.01.

*** *p* < 0.001.

Using bootstrap sampling with bias correction, the total mediation effect was examined. As shown in Table 5, the total mediation effect value was −0.040, with the 95% bootstrap confidence interval not containing zero. This indicates that the total mediation effect of trait mindfulness on rumination among college students is significant. The total mediating effect comprises three pathways: Ind1: “trait mindfulness → exercise behaviour → rumination” with an effect value of −0.005; Ind2: “trait mindfulness → optimistic intelligence quotient → rumination” with an effect value of −0.003; Ind3: The effect value of “trait mindfulness → exercise behaviour → optimistic intelligence quotient → rumination” is −0.093. Among these, the Bootstrap 95% confidence interval does not include 0, indicating that the chained mediating effect is significant. The results of the three paths respectively validate Hypothesis 2, Hypothesis 3, and Hypothesis 4.

**Table 5:** Mediated effects test.

Trails	Efficiency value	Boot SE	95% confidence interval	
			BootLLCI	BootULCI
Total effect	−0.021	0.007	−0.035	−0.006
Direct effect	−0.013	0.008	−0.028	0.002
Path 1	−0.005	0.005	−0.025	−0.006
Path 2	−0.003	0.004	−0.016	−0.002
Path 3	−0.093	0.025	−0.143	−0.044
Total indirect effect	−0.040	0.012	−0.063	−0.018

## Discussion

To explore the underlying mechanism through which trait mindfulness influences rumination among college students, this study constructed and validated a chain mediation model linking trait mindfulness to rumination by introducing exercise behaviour and optimistic intelligence quotient as mediating variables. The findings reveal the internal mechanism by which trait mindfulness affects rumination in college students, while also providing empirical evidence for improving rumination and promoting mental health among this population.

### Relationship between trait mindfulness and rumination

This study found a significant negative correlation between trait mindfulness and rumination among college students. After controlling for variables and incorporating mediating variables, trait mindfulness significantly predicted rumination negatively, indicating that higher levels of trait mindfulness correlate with lower rumination levels. Hypothesis H1 was thus validated. The trait mindfulness coping model posits that mindfulness reduces rumination by altering individuals' responses to negative emotions and thoughts (8), thereby improving mental health (7). It also helps individuals recognize and interrupt rumination patterns, enhancing acceptance and regulation of negative emotions to effectively lower anxiety levels (10). This model provides theoretical support for understanding mindfulness's role in rumination. Research indicates that trait mindfulness, as a psychological process focused on nonjudgmental awareness of the present moment, can help curb rumination (11). In summary, these findings align with previous research (10, 11), confirming that trait mindfulness effectively reduces rumination levels among college students.

### The mediating role of exercise behavior

Mediating variables are crucial for explaining how trait mindfulness influences college students' rumination. This study constructed a mediation model of trait mindfulness influencing rumination in college students, with exercise behaviour and optimistic intelligence quotient as mediating variables. The finding that exercise behaviour mediates the relationship between trait mindfulness and rumination indicates that mindfulness negatively predicts rumination in college students (17) while also indirectly influencing rumination levels through exercise behaviour. Related research on the relationship between mindfulness and health behaviours reveals that mindfulness positively promotes health behaviours (15). Studies indicate that trait mindfulness can effectively enhance exercise behaviour through state mindfulness and intentional self-regulation, with higher trait mindfulness levels correlating with stronger exercise motivation (13). For instance, mindfulness-based recovery therapies can top-down improve unhealthy lifestyles like alcohol abuse and promote exercise behaviour through cognitive control. Trait mindfulness enables exercisers to reach higher exercise stages (16). Furthermore, engaging in moderate physical exercise can effectively reduce an individual's rumination.

### The mediating role of optimistic intelligence quotient

Optimistic intelligence quotient is not merely an attitude but a capability—a power to influence or alter an individual's future. It is a factor more decisive of personal destiny than IQ or EQ (35). As a positive intervention method that enhances optimistic intelligence quotient, mindfulness training helps develop effective emotional regulation strategies (24), enabling individuals to face negative events with greater optimism and confidence (22). Extensive research has promoted mindfulness training as a positive intervention to enhance physical and mental health and well-being across schools, families, and communities. Implementing mindfulness training in education can elevate college students' optimistic intelligence quotient levels and foster a positive, optimistic learning environment (23). Furthermore, college students with high optimistic intelligence quotient are adept at deriving positive emotions from negative events (20) and recover more quickly from setbacks (21).

### Chain mediation effect of trait mindfulness on rumination

The Bootstrap method validated the chain mediation effect of exercise behaviour and optimistic intelligence quotient in trait mindfulness's negative influence on college students' rumination. The constructed chain mediation model offers new perspectives for further advancing and understanding the relationship between trait mindfulness and rumination among college students. Research indicates that exercise behaviour positively influences college students' optimistic intelligence quotient, contributing to its enhancement (28). As exercise duration and frequency increase, students’ optimistic intelligence quotient levels steadily rise. Exercise behaviour serves as both the mediating variable and the core value through which optimistic intelligence quotient promotes directional mental health development (25), exhibiting a significant positive predictive effect on college students' optimistic intelligence quotient (26). This stems from the fact that engaging in exercise behaviour allows college students to experience overcoming physical fatigue and challenging themselves, thereby enhancing their stress resilience. This fosters positive thinking and emotions, leading to heightened positive affective experiences (30). Evidently, exercise behaviour plays a significant role in cultivating college students' optimistic intelligence quotient.

### Limitations and future prospects

This study examines the relationship between trait mindfulness and rumination among college students. The constructed chain mediation model reveals the underlying mechanism through which trait mindfulness influences rumination in university settings, offering new perspectives and insights. This holds significant theoretical and practical implications for reducing rumination among college students. However, the study also has several limitations:

(1) First, the study sample consisted of college students from selected universities in Liaoning and Henan provinces, excluding students from other regions and other groups. The representativeness and generalizability of the findings require further expansion. Future research should broaden the scope by recruiting a more diverse sample to examine the applicability of these conclusions. Additionally, the use of convenience sampling may introduce bias risks. Liaoning and Henan provinces represent regions with moderate economic development. Student traits such as trait mindfulness, exercise behaviour, optimistic intelligence quotient, and rumination levels may differ in regions with higher or lower economic development. This could potentially affect the universality of the findings.
(2) Secondly, this cross-sectional design limits the ability to establish causal relationships and restricts interpretations of variable interactions. For instance, while trait mindfulness was found to negatively predict rumination among college students—consistent with prior research—other latent variables may have influenced this relationship. Moving forward, the team will conduct longitudinal tracking studies to further explore the long-term influence mechanisms of trait mindfulness on college students' rumination, thereby scientifically and accurately revealing causal relationships between variables.
(3) Third, while gender and grade level were controlled as variables in testing relationships, other uncontrolled confounding variables may exist beyond these factors. For instance, research indicates that varying levels of stress among college students can increase rumination. Given differences in academic stages, life pressures, and stress resilience among students, these factors may influence rumination patterns and consequently affect the relationship between trait mindfulness and rumination. Future research will comprehensively analyze variables that could impact findings, enhancing scientific rigor through strict control measures.
(4) Finally, it must be acknowledged that some effect sizes in this study were relatively small, potentially reflecting the complexity of psychological variable interactions. The formation and alleviation of rumination among college students result from multiple factors. Beyond the exercise behaviour and optimistic intelligence quotient included in this study, unaccounted variables such as academic pressure, parent-child relationships, and coping styles may have diluted the independent effects of trait mindfulness and single mediating variables. This limitation suggests that future research should construct more comprehensive variable models incorporating additional potential factors. This approach would more fully elucidate the multifaceted mechanisms through which trait mindfulness influences rumination while also enabling clearer assessment of the true magnitude of path effects.

## Conclusion

Trait mindfulness significantly and negatively predicted rumination among college students. Both the direct and indirect effects of trait mindfulness on rumination were significant. The indirect effects encompassed the independent mediating roles of exercise behaviour and optimistic intelligence quotient, as well as their chain-mediated effects.

## Data Availability

The raw data supporting the conclusions of this article will be made available by the authors, without undue reservation.
